# Impacts of the Glucuronidase Genotypes *UGT1A4*, *UGT2B7*, *UGT2B15* and *UGT2B17* on Tamoxifen Metabolism in Breast Cancer Patients

**DOI:** 10.1371/journal.pone.0132269

**Published:** 2015-07-15

**Authors:** Alicia Romero-Lorca, Apolonia Novillo, María Gaibar, Fernando Bandrés, Ana Fernández-Santander

**Affiliations:** 1 Department of Basic Biomedical Sciences, Faculty of Biomedical Sciences, Universidad Europea de Madrid, Villaviciosa de Odón, Madrid, Spain; 2 School of Advanced Studies, Fundación Tejerina, Madrid, Spain; IPO, Portuguese Institute of Oncology of Porto, PORTUGAL

## Abstract

Tamoxifen is used to prevent and treat estrogen-dependent breast cancer. It is described as a prodrug since most of its antiestrogen effects are exerted through its hydroxylated metabolites 4-OH-tamoxifen and endoxifen. In prior work, we correlated optimal plasma levels of these metabolites with certain genotypes of *CYP2D6* and *SULT1A2*. This descriptive study examines correlations between concentrations of tamoxifen's glucuronide metabolites and genotypes *UGT1A4 Pro24Thr*, *UGT1A4 Leu48Val*, *UGT2B7 His268Tyr*, *UGT2B15 Asp85Y*Tyr *UGT2B15 Lys523Thr *and *UGT2B17del *in 132 patients with estrogen receptor-positive breast cancer under treatment with tamoxifen. Patients were genotyped by real-time and conventional PCR-RFLP. The glucuronides 4-OH-tamoxifen-N-glucuronide, 4-OH-tamoxifen-O-glucuronide and endoxifen-O-glucuronide were isolated from blood plasma and quantified using a high-pressure liquid chromatography-tandem mass spectrometry system. Individuals who were homozygous for *UGT1A4^48VAL^* showed significantly lower mean concentrations of both glucuronide metabolites compared to subjects genotyped as wt/wt plus wt/48Val (p=0.037 and p=0.031, respectively). Women homozygous for UGT2B7^268Tyr^ also showed mean substrate/product ratios of 4-OH-tamoxifen/4-OH-tamoxifen-O-glucuronide and 4-OH-tamoxifen/4-OH-tamoxifen-N-glucuronide indicative of reduced glucuronidase activity compared to wt homozygotes or to heterozygotes for the polymorphism (p=0.005 and p=0.003, respectively). In contrast, *UGT2B15 Lys523Thr *and *UGT2B17del* were associated with possibly increased enzyme activity. Patients with at least one variant allele *UGT2B15^523Thr^* showed significantly higher 4-OH-tamoxifen-O-glucuronide and endoxifen-glucuronide levels (p=0.023 and p=0.025, respectively) indicating a variant gene-dose effect. Higher 4-OH-tamoxifen-N-glucuronide levels observed in *UGT2B17del* genotypes (p=0.042) could be attributed to a mechanism that compensates for the greater expression of other genes in *UGT2B17* del/del individuals. Our observations suggest that patients carrying mutations *UGT1A4^48Val^*, *UGT2B7^268Tyr^* or with wt genotypes for *UGT2B17^nodel^* and *UGT2B15^523Lys^* could be the best candidates for a good response to tamoxifen therapy in terms of eliciting effective plasma active tamoxifen metabolite levels. However, additional studies examining the effects of UGT genotype on overall patient response to TAM are needed to further examine the role of UGT polymorphisms in the therapeutic efficacy of TAM.

## Introduction

Tamoxifen (TAM) is a nonsteroid antiestrogen used for the treatment and prevention of estrogen-dependent breast cancer (BC) [1]. TAM is metabolized via the cytochrome P450-mediated pathway to several primary and secondary metabolites that show variable potency towards the estrogen receptor. TAM is described as a pro-drug since two of its metabolites 4-hydroxy-tamoxifen (4-OH-TAM) and N-desmethyl-4-hydroxy-tamoxifen (endoxifen) both have an affinity for the estrogen receptor that markedly exceeds that of TAM itself [2]. Both 4-OH-TAM and endoxifen are produced by *CYP2D6*-mediated phase I metabolism, and a relationship between *CYP2D6* genetic variability and plasma concentrations of these two metabolites has been reported by several authors [3, 4, 5, 6].

The main routes of elimination of TAM and its metabolites are via phase II conjugation reactions including sulfation and glucuronidation. Sulfotransferase enzymes are a family of phase II liver enzymes that detoxify a variety of xenobiotic and endogenous compounds. These enzymes catalyze the transfer of a sulfonyl group to nucleophilic groups increasing their solubility and facilitating their excretion. In prior work, we observed a possible benefit of carriers of alleles that lead to lower enzyme activity levels (SULT1A2*2 and SULT1A2*3) in maintaining optimal levels of 4-OH-TAM and endoxifen [6]. Detoxification of TAM and its metabolites also occurs via glucuronidation. Glucuronidation process takes place in the liver by TAM conjugation to glucuronic acid [7]. TAM glucuronide conjugates were determinated in urine and plasma samples of breast cancer patients treated with this drug [8,9]. Nowell et al. [10] also considered glucuronidation process within target tissues such as the adipose tissue of the breast.

Both TAM and 4-OH-TAM undergo N-glucuronidation, whereas O-glucuronidation has been observed for 4-OH-TAM and endoxifen [11, 12]. The enzyme UGT1A4 catalyzes the formation of a quaternary ammonium-linked glucuronide with TAM [13, 14], and this enzyme action is also observed on a variety of carcinogenic compounds, androgens, progestins and plant steroids [15, 16, 17]. Two unlinked missense polymorphisms have been identified at codon 24 Pro>Thr (rs6755571) and codon 48 Leu>Val (rs2011425) within this gene [18]. The impacts of these two mutations on rates of glucuronidation depend on the substrate. The findings of *in vitro* studies indicate similar TAM glucuronidation rates between both mutant variants (*UGT1A4* ^*24Thr*^ and *UGT1A4* ^*48Val*^) and the wild type enzyme [11, 19]. However, the codon 24 polymorphism has been linked to significantly greater glucuronidation activity against the tobacco-specific nitrosamine [20], while the *UGT1A4* ^*48Val*^ variant gives rise to decreased rates of glucuronidation against dihydrotestosterone after transient transfection into cell lines *in vitro* [18]. The major hepatic isoform UGT2B17 has over 95% amino acid sequences in common with *UGT2B15* and shows similar substrate specificity. This high sequence identity between UGT2B15 and UGT2B17 suggests that the genes arose from a duplication event [21]. Wilson et al. [22] reported the variable frequency among different ethnic groups of the absence of a 150 kb genomic interval spanning the entire *UGT2B17* gene. No effects of this *UGT2B17* deletion have been detected on the availability of oxazepam and androgenic steroid substrates [23, 24]. However, the influence of the gene deletion on TAM metabolites has not yet been addressed.

*UGT2B15* was originally identified as important for the glucuronidation of androgenic steroids [25]. However, subsequent studies have also indicated its role in the metabolism of drugs, drug metabolites and other xenobiotics [26]. Two common non-synonymous polymorphisms in the *UGT2B15* gene, Asp85Tyr (rs1902023) and Lys523Thr (rs4148269) appear to influence *UGT2B15* S-oxazepam activity in human liver microsomes (HLM)[27]. However, it is unlikely that *UGT2B15* ^*Asp85Tyr*^ determines interindividual variation in TAM metabolites because no significant differences have been observed among genotypes in plasma metabolite concentrations [28]. There are no data describing the effects of the *UGT2B15* ^*Lys523Thr*^ polymorphism on TAM metabolism though conflicting results regarding glucuronidation activity determining oxazepam availability have been reported [23, 27]. UGT2B7 is the main liver enzyme responsible for O-glucuronidation of the trans isomers of 4-OH-TAM and endoxifen [11]. In a prevalent missense polymorphism identified for the *UGT2B7* gene, tyrosine replaces histidine at amino acid 268 (rs 7439366) [29]. However, despite no correlation detected between plasma concentrations of endoxifen or 4-OH-TAM and the *UGT2B7* ^*268Tyr*^ variant by some authors [28], others report a significant trend in decreasing 4-OH-TAM O-glucuronidation with increasing numbers of *UGT2B7* ^*268Tyr*^ alleles in both HLM [30] and cell lines [29].

Given the complexity of TAM metabolism and the scarce information on this topic in the literature, this descriptive study examines relationships between TAM glucuronide metabolite concentrations and genotypes for *UGT1A4* Pro24Thr, *UGT1A4* Leu48Val, *UGT2B7* His268Tyr, *UGT2B15* Asp85Tyr, *UGT2B15* Lys523Thr, *UGT2B17del* in 132 Spanish patients with estrogen receptor-positive breast cancer under tamoxifen treatment. Our previous findings indicated that besides the *CYP2D6* genotype, *SULT1A2* also seems to play a role in maintaining optimal levels of both 4-hydroxy-tamoxifen and endoxifen [6]. Besides, this paper presents the first data on the effects of *UGT2B17* and *UGT2B15 Lys523Thr* genotypes on plasma TAM glucuronide metabolite levels.

## Patients and Methods

### Ethics Statement

The study protocol was approved by the Review Board of the Hospital de Getafe (Madrid, Spain). Written informed consent to participate in the study was obtained from all participants.

### Patients

One hundred and thirty two Caucasian patients of Spanish descent recently diagnosed with ER positive breast tumors were recruited from the *Fundación Tejerina-Centro de Patología de la Mama* (Madrid). Premenopausal and postmenopausal women were enrolled when started on TAM as standard adjuvant therapy after undergoing primary surgery, radiation and adjuvant chemotherapy. Patients were excluded if they had started TAM therapy simultaneously with either adjuvant chemotherapy or adjuvant radiation therapy (or both) or if they were undergoing other adjuvant endocrine therapies. Patients who were pregnant or breast-feeding were also excluded from the study. Enrolled patients were allowed to take vitamin E, selective serotonin reuptake inhibitors (SSRIs) or herbal remedies. Blood samples were collected within 3 to 60 months of initiating TAM treatment.

### Samples

Venous blood was collected from all 132 subjects before taking their daily 20-mg dose of TAM. Ten milliliters of heparin plasma were separated by centrifugation and immediately stored at -20°C until analysis. In addition, one milliliter of an EDTA blood sample was taken for subsequent DNA extraction and genotyping. DNA was isolated from peripheral leukocytes using a QIAamp DNA Blood Mini Kit (Qiagen, Madrid, Spain) according to the manufacturer’s instructions. The DNA concentration was determined and adjusted to 2–20 ng/μL.

### Genotyping

The *UGT2B7 His268Tyr* variant allele at codon 268 (rs7439366) was determined by PCR-RFLP using the primers described by Wiener et al. [20] and the *Fok*I enzyme. The SNPs *UGT1A4 Pro24Thr* (rs 6755571) and *UGT1A4 Leu48Val* (rs2011425) were genotyped using two pairs of primers and the *HPY*188III and *Stu*I enzymes, respectively, by PCR-RFLP as described by Wiener et al. [20]. To determine the presence or absence of the *UGT2B17* allele deletion, the gene was amplified as described by He et al. [23] with two pairs of primers. The presence/absence of amplification with each pair of primers determined the genotype wild type (non-del) or mutant (del). SNPs *UGT2B15 Asp85Tyr* (rs1902023) and *UGT2B15 Lys523Thr* (rs4148269) were assessed by qPCR as described by He et al. [23].

Any mutations detected were confirmed by repeating the procedure or using a different technique whenever possible. Samples were discarded if there was disagreement between the methods or repetitions.

### Quantifying tamoxifen and its metabolites in plasma

#### Reagents and chemicals

4-hydroxy-tamoxifen-N-ß-D-glucuronide (1:1 E/Z mixture) (4-OH-TAM-N-Gluc), 4-hydroxy-tamoxifen-O-ß-D-glucuronide (E) (4-OH-TAM-O-Gluc), endoxifen-O-ß-D-glucuronide (1:1 E/Z mixture) (endoxifen-Gluc), 4-hydroxy-tamoxifen-d5, N-desmethyl-4-hydroxy-tamoxifen-d5 (1:1 E/Z mixture) and tamoxifen-d5-N-ß-D-glucuronide (1:1 E/Z mixture) were purchased from Toronto Research Chemicals (North York, Ontario, Canada).

Acetonitrile, methanol, distilled water and formic acid were obtained from Fluka Analytical (Sigma-Aldrich, Spain). All chemicals used were of analytical grade. Small (1 mL) volumes of drug-free human serum were pooled and used for validation purposes.

#### HPLC

TAM and its metabolites were separated and quantified by high-pressure liquid chromatography-tandem mass spectrometry using an Agilent HPLC 1200 system. HPLC experiments were performed using a binary pump G1312A, a G1316A column oven, G1379B degasser and an automatic injector H-ALS G1367B. Mobile phases A and B consisted of ammonium formate 3.5 mM, pH 3.5, and acetonitrile, respectively. Mobile phases A and B were pumped through an ACE C-18 precolumn (10 mm x 2.1 mm I.D., 3.5 μm Aglilent USA) and a ZORBAX Eclipse XDB-C18 column (150 mm x 2.1 mm I.D., 3.5 μm, Agilent USA) at a flow rate of 0.2 mL/min using the gradient shown in Table 1. Separation was conducted at 40°C. Aliquots (22-μL) were injected and the autosampler needle was rinsed in acetonitrile/water solution (1:1). The total run time was 20 min. During the first 4.0 and last 2.0 min, the eluent was removed using a diverter valve to avoid endogenous compounds entering the mass spectrometer.

**Table 1:** Parameters of the HPLC gradient used to separate tamoxifen and its metabolites on a ZORBAX Eclipse XDB-C18 column (150 mm x 2.1 mm I.D., 3.5 μm) at 40°C.

Time (minutes)	Flow rate (mL/min)	Mobile phase A^a^ (%)	Mobile phase B^b^ (%)
0	0.2	60	40
3	0.2	50	50
6	0.2	10	90
11	0.2	60	40
14	0.2	60	40

^a^ Mobile phase A: ammonium formate 3.5 mM pH = 3.5

^b^ Mobile phase B: acetonitrile

As detector, we used an A 6410 Triple Quadrupole mass spectrometer (Agilent Technologies, USA) equipped with a heated electrospray ionization source (Thermo Fisher Scientific, Waltham, MA, USA) operating in positive ion mode. For quantification, multiple reaction monitoring (MRM) chromatograms were acquired using Mass Hunter software version B.01.04 (Agilent Technologies, USA). Positive ions were created at atmospheric pressure. Quadrupoles were operated at unit resolution (0.7 Da). The HESI/MS/MS operating parameters and mass transitions are provided in Table 2.

**Table 2:** HPLC and MS parameters used to discriminate glucuronide tamoxifen metabolites.

Tamoxifen metabolite	Precursor ion (amu)	Product ion (amu)	Dwell (ms)	Fragmentor (v)	Collision energy (v)	Retention time (min)	MRM^a^	LLOQ^b^ (nM)
N-desmethyl-4OH-tamoxifen- ß-D- glucuronide	550	375.2	100	152	19	2.18	550 → 375.2	0.004
4-OH-tamoxifen-O-ß- D-glucuronide (E)	564	388	100	176	23	2.29	564 → 388	0.007
4-OH-tamoxifen-N-ß-D-glucuronide (E/Z)	565	389.2	100	152	19	2,26	565 → 389.2	0.008

^a^ Multiple reaction monitoring

^b^ Lower limit of quantification

#### Calibration standards, quality controls and internal standard solutions

Two separate stock solutions of all analytes (1 mg/mL) and internal standards (1 mg/mL) were prepared by dissolving accurately-weighed approximate 1 mg amounts in 1 mL of methanol. One stock solution was used to prepare calibration standards and the other stock solution to prepare quality control standards. A mixture of internal standard stock solutions was prepared and this mixture was diluted in acetonitrile to obtain a working solution for sample pretreatment. This internal standard working solution contained: 4-hydroxy-tamoxifen-d5, N-desmethyl-4-hydroxy-tamoxifen-d5 (1:1 E/Z mixture) and tamoxifen-d5 N-ß-D-glucuronide (1:1 E/Z mixture).

#### Sample preparation

A 280-μL volume of 1% formic acid in methanol containing internal standards was added to a 100-μL serum aliquot. After vortexing and centrifugation, the clear supernatant was transferred to a HybridSPE column (Supelco) and the eluents stored at 2–8°C until analysis. Samples were analyzed in triplicate.

#### Validation procedures

Eleven non-zero calibration standards were prepared in duplicate for each run and analyzed in three independent runs. Calibration curves (area ratio obtained with the internal standard versus nominal concentration) were fitted by least-squares linear regression using the reciprocal of the squared concentration (1/x2) as a weighting factor. The method's intra- and inter-assay accuracy and precision were determined by assaying three replicates of each of the quality control samples at the lower limit of quantification (LLQ), at a low, medium and a high concentration level in three separate runs. The concentrations of each quality control sample were calculated using the calibration standards that were analyzed in duplicate in the same run. Differences between nominal and measured concentrations were used to calculate accuracy. Accuracy should be within 85–115% and precision should not exceed 15% of the coefficient of variation. Carry-over was determined by injecting a processed control human serum sample after an upper limit of quantification sample. Areas of peaks in the blank processed sample should be within 20% of the peak area of the LLQ sample. Four individual batches of control human serum were used to assess the specificity and selectivity of the method. To determine whether endogenous constituents interfere with the assay, a double blank and a sample spiked at the LLQ were processed from these batches.

Tamoxifen, 4-OH-tamoxifen, N-desmethyl-tamoxifen, N-desmethyl-4-OH-tamoxifen (1:1 E/Z mixture) and tamoxifen-N-oxide concentrations were determined as described elsewhere [6].

### Data analysis

Genotype frequencies, allele frequencies and Hardy-Weinberg equilibria were determined using the Genepop software package (v 4.1). Descriptive statistics were calculated using standard methods. Metabolic ratios were calculated as the concentration of substrate/concentration of metabolite. The non-normal distribution of data was confirmed by the Kolmogorov-Smirnov test. Levels of TAM, its metabolites and ratios were compared among genotype groups using Wilcoxon-Mann-Whitney or Kruskal-Wallis tests. All statistical tests (two-sided) were performed using SPSS software (version 18.0 SPSS, Chicago, IL). Significance was set at a p < 0.05.

## Results

### Demographics

The cohort examined was comprised of 132 Spanish patients with BC from different geographic regions. Mean age was 53.12 years (SD = 9.87, range 30 to 81 years). Tumor types were: 51.7% infiltrating ductal carcinoma, 29.9% ductal carcinoma *in situ*, 11.5% infiltrating lobular carcinoma and 6.9% both infiltrating ductal carcinoma and ductal carcinoma *in situ*.

### Genotyping

*UGT1A4*, *UGT2B7*, *UGT2B15* and *UGT2B17* genotype and allele frequencies are provided in Tables 3 and 4, respectively. Allelic frequencies of the *UGT1A4* ^*24Thr*^ and *UGT1A4* ^*48Val*^ mutant variants, 7% and 6% respectively, are within the ranges reported for other Caucasians populations [18, 20, 31] but lower than those found in Japanese subjects [32]. The prevalence of the *UGT2B17* deletion allele was 26% in our sample. This frequency is within the reported range for other populations despite variation observed among ethnic groups, e.g., rates of 21% and 33% respectively have been provided for African Americans and Caucasians [22]. Frequencies of the polymorphisms *UGT2B15* ^*Asp85Tyr*^ and *UGT2B15* ^*Lys523Thr*^ were 48% and 44%, respectively, in line with data for other European populations [33, 27]. The variant allele *UGT2B7* ^*268Tyr*^ was detected in 31% of our patient population. This polymorphism shows a high prevalence affecting 54.4% of persons in the UK or 46% of the general Caucasian population [34, 33]. All the UGT gene frequencies obtained exhibited good agreement with Hardy-Weinberg equilibrium. The data for some patients (<3%) were eliminated due to conflicting genotyping results.

**Table 3:** *UGT1A4*, *UGT2B7*, *UGT2B15* and *UGT2B17* genotype frequencies. International codes for SNPs between brackets.

GENOTYPE (sample size)	FREQUENCY (%)
***UGT1A4 Pro24Thr*** [rs 6755571] (n = 130)	
*UGT1A4* ^*24Pro*^ */UGT1A4* ^*24Pro*^ (wt/wt)	86.9
*UGT1A4* ^*24Pro*^ */UGT1A4* ^*24Thr*^	12.3
UGT1A4^24Thr^ / UGT1A4^24Thr^	0.8
***UGT1A4 Leu48Val*** [rs2011425] (n = 130)	
*UGT1A4* ^*48Leu*^ */UGT1A4* ^*48Leu*^ (wt/wt)	90.0
*UGT1A4* ^*48Leu*^ */UGT1A4* ^*48Val*^	8.5
*UGT1A4* ^*48Val*^ */UGT1A4* ^*4Val*^	1.5
***UGT2B7 His268Tyr*** [rs7439366] (n = 125)	
*UGT2B7* ^*268His*^ */ UGT2B7* ^*268His*^ (wt/wt)	60.0
*UGT2B7* ^*268His*^ */ UGT2B7* ^*268Tyr*^	17.6
*UGT2B7* ^*268Tyr*^ */ UGT2B7* ^*268Tyr*^	22.4
***UGT2B15 Asp85Tyr*** [rs1902023] (n = 132)	
*UGT2B15* ^*85Asp*^ */UGT2B15* ^*85Asp*^ (wt/wt)	29.5
*UGT2B15* ^*85Asp*^ */UGT2B15* ^*85Tyr*^	44.0
*UGT2B15* ^*85Tyr*^ */UGT2B15* ^*85Tyr*^	26.5
***UGT2B15 Lys523Thr*** [rs4148269] (n = 127)	
*UGT2B15* ^*523Lys*^ */ UGT2B15* ^*523 Lys*^ (wt/wt)	29.2
*UGT2B15* ^*523Lys*^ */ UGT2B15* ^*523Thr*^	53.5
*UGT2B15* ^*523Thr*^ */ UGT2B15* ^*523Thr*^	17.3
***UGT2B17*** (n = 131)	
wt/wt	57.3
wt/del	33.6
del/del	9.1

**Table 4:** *UGT1A4*, *UGT2B7*, *UGT2B15* and *UGT2B17* allele frequencies. International codes for SNPs provided in brackets.

ALLELE (individuals)	FREQUENCY (%)
***UGT1A4 Pro24Leu*** [rs 6755571] (n = 130)	
*UGT1A4* ^*24Pro*^ (wt)	93.0
*UGT1A4* ^*24Thr*^	7.0
***UGT1A4 Leu48Val*** [rs2011425] (n = 130)	
*UGT1A4* ^*48Leu*^ (wt)	94.0
*UGT1A4* ^*48Val*^	6.0
***UGT2B7 His268Tyr*** [rs7439366] (n = 125)	
*UGT2B7* ^*268His*^ (wt)	69.0
*UGT2B7* ^*268Tyr*^	31.0
***UGT2B15 Asp85Tyr*** [rs1902023] (n = 132)	
*UGT2B15* ^*85Asp*^ (wt)	52.0
*UGT2B15* ^*85Tyr*^	48.0
***UGT2B15 Lys523Thr*** [rs4148269] (n = 127)	
*UGT2B15* ^*523Lys*^ (wt)	56.0
*UGT2B15* ^*523Thr*^	44.0
***UGT2B17del*** (n = 131)	
wt	74.0
del	26.0

### Plasma concentrations of tamoxifen and its metabolites

The mean TAM concentration of the samples analyzed was 203.07 ± 95.70 ng/mL. Mean NDM-TAM was over 2 fold the TAM concentration (451.93 ± 191.05 ng/mL). Among TAM’s known clinically active hydroxylated metabolites, endoxifen showed the highest mean concentration at three times the mean 4OH-TAM concentration (24.57 ± 19.52 ng/mL *vs*. 7.87 ± 7.33 ng/mL). Mean concentrations of the glucuronide metabolites 4-OH-TAM-N-Gluc, 4-OH-TAM-O-Gluc and endoxifen-O-Gluc were 21.62 ± 16.08 ng/mL, 5.21 ± 4.58 ng/mL and 20.56 ± 15.53 ng/mL, respectively. TAM-N-ß-D-glucuronide could not be quantified because of its low plasma concentrations. The data for some patients (<3%) were eliminated because of conflicting results or technical problems.

### Correlating UGT genotypes with plasma TAM metabolite concentrations

**Fig 1:**
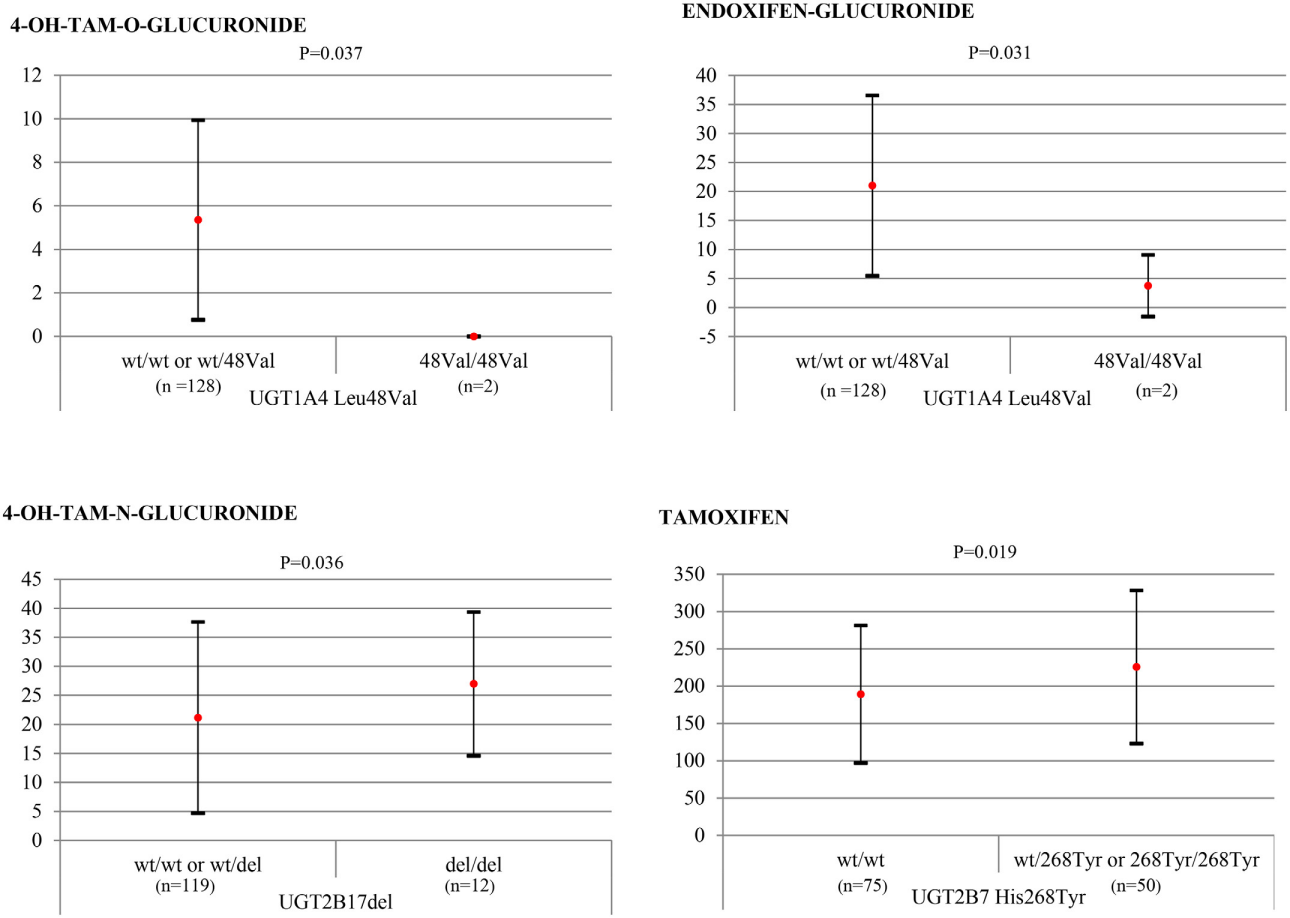
Concentrations of tamoxifen metabolites (means ± standard deviations, ng/mL) by *UGT1A4* ^*Leu48Val*^ and *UGT2B17* ^*del*^ genotype subgroups established according to wt allele doses. Sample sizes indicated in brackets.

**Fig 2:**
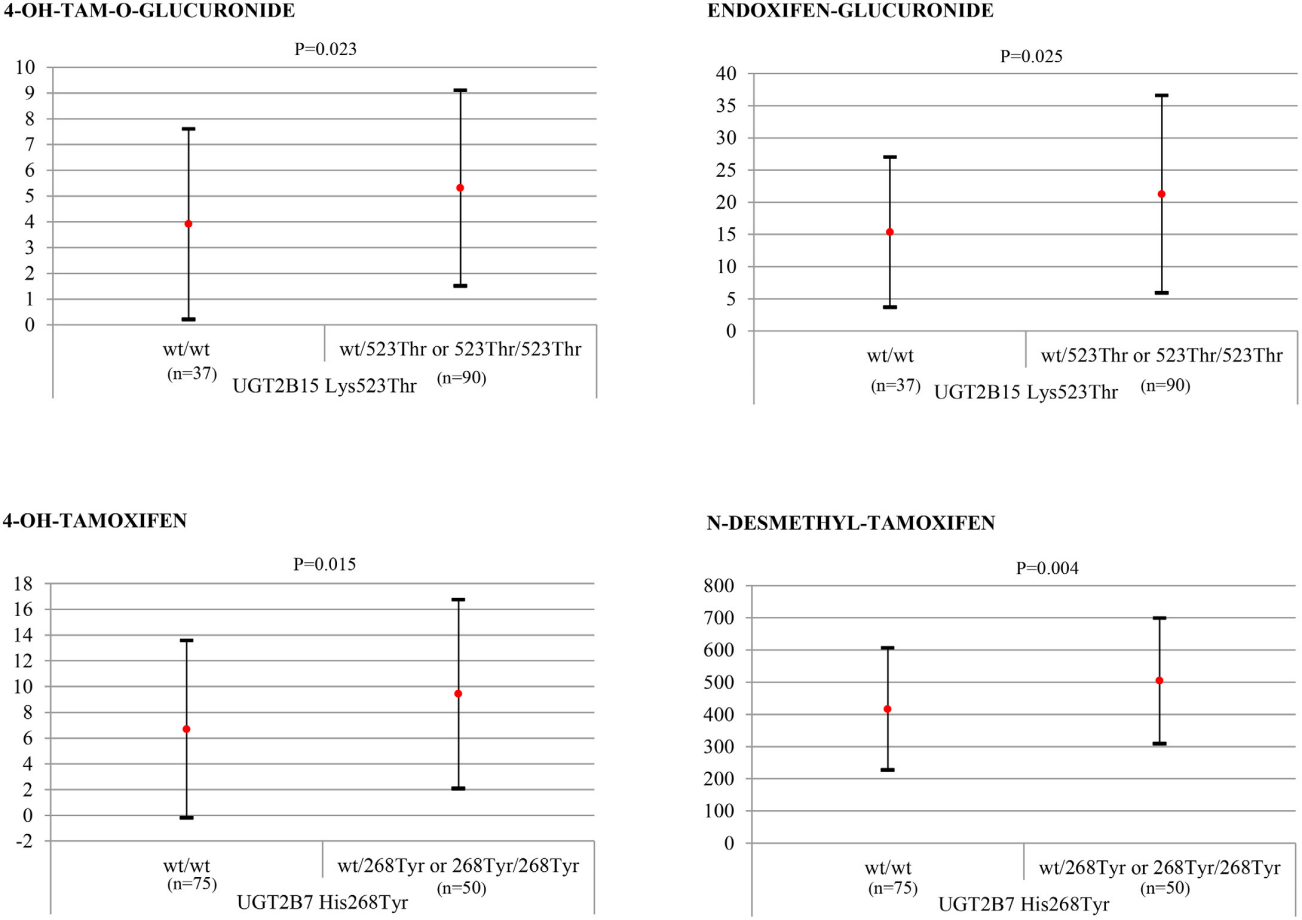
Concentrations of tamoxifen metabolites (mean ± standard deviation, ng/mL) by *UGT2B15* ^*Lys523Thr*^ and *UGT2B7* ^*His268Tyr*^ genotype subgroups established according to wt allele doses. Sample sizes indicated in brackets.

In Table 5 we provide the means, standard deviations, medians and ranges of the concentrations of TAM and its metabolites recorded for the *UGT1A4*, *UGT2B7*, *UGT2B15* and *UGT2B17* genotypes. No significant differences in TAM metabolite concentrations were observed between the two *UGT1A4* genotypes (SNPs *UGT1A4* ^24THR^ and *UGT1A4* ^48VAL^) (Table 5). However, patients homozygous for *UGT1A4* ^48VAL^ showed significantly lower mean concentrations of both of 4-OH-TAM-O-Gluc and endoxifen-Gluc compared to wt/wt plus wt/48Val patients (p = 0.037 and p = 0.031, respectively, Fig 1). Significant variation in mean 4-OH-TAM-N-Gluc levels was observed among the three *UGT2B17* genotypes (wt/wt, del/del, wt/del) (p = 0.042, Table 5) and between the groups del/del versus wt/wt plus wt/del (p = 0.036, Fig 1). The mean 4-OH-TAM/4-OH-TAM-N-Gluc ratio was significantly lower in del/del patients versus remaining patients (0.20 ± 0.18 and 0.5 ± 0.73, p = 0.028). No significant differences in mean plasma metabolite concentrations were observed between the two *UGT2B15* genotypes (*Asp85Tyr* and *Lys523Thr)* (Table 5). However, for *UGT2B15 Lys523Thr*, differences among genotypes in plasma 4-OH-TAM-O-Gluc and endoxifen-Gluc levels approached significance (0.07 and 0.082, respectively, Table 5). When genotypes groups were established for *Lys523Thr* (wt/wt *versus* wt/523Thr + 523Thr/523Thr) a variant gene-dose effect was observed; patients with at least one variant allele showed significantly higher mean levels of 4-OH-TAM-O-Gluc and endoxifen-Gluc (0.023 and 0.025, respectively, Fig 2). *UGT2B7* genotypes featured significantly varying mean plasma levels of TAM, 4-OH-TAM and N-desmethyl-TAM (p = 0.049, p = 0.006 and p = 0.012 respectively, Table 5). Significant differences in the same metabolites also emerged when genotypes were grouped as wt/wt + wt/268Tyr versus 268Tyr/268Tyr or wt/wt versus wt/268Tyr + 268Tyr/268Tyr (Fig 2). The substrate/product ratios determined, 4-OH-TAM/4-OH-TAM-O-Gluc and 4-OH-TAM/4-OH-TAM-N-Gluc, indicated significantly reduced activity of the enzyme in 268Tyr/268Tyr individuals both in the separate genotype (p = 0.014 and p = 0.011, respectively) and grouped genotype (p = 0.005 and p = 0.003, respectively) comparisons.

**Table 5:** Concentrations (ng/mL) of tamoxifen metabolites (means (±SD), medians (in cursive) and ranges (in brackets)) detected in patients according to their *UGT1A4*, *UGT2B7*, *UGT2B15* and *UGT2B17* genotypes.

Genotype	4-OH-tamoxifen N-ß-D-glucuronide	4-OH-tamoxifen O-ß-D-glucuronide	Endoxifen ß-D-glucuronide	Tamoxifen	4-OH-tamoxifen	N-desmethyl-tamoxifen	Endoxifen	Tamoxifen-N-oxide
***UGT1A4 Pro24Thr*** wt/wt (n = 113)	20.33 ± 11.44 *17*.*87* [0.0–64.51]	4.85 ± 3.88 *4*.*53* [0.0–18.97]	20.21 ± 15.28 *16*.*84* [0.0–79.93]	200.11 ± 98.09 *170*.*83* [77.10–576.23]	7.88 ± 7.50 *6*.*11* [0.0–35.90]	448.40 ± 196.83 *399*.*99* [35.96–1,210.09]	24.79 ± 19.72 *19*.*38* [0.0–107.10]	49.64 ± 23.68 *45*.*33* [15.69–142.97]
***UGT1A4 Pro24Thr*** wt/24Thr (n = 16)	31.45 ± 34.27 *21*.*49* [11.83–153.88]	7.77 ± 7.93 *5*.*61* [0.0–34.42]	23.86 ± 18.70 *18*.*25* [0.0–73.72]	214.97 ± 74.67 *190*.*69* [115.35–371.37]	8.66 ± 7.04 *6*.*53* [0.0–28.84]	443.04 ± 142.35 *474*.*54* [228.81–638.82]	24.59 ± 20.78 *16*.*39* [4.18–74.06]	55.29 ± 23.86 *52*.*30* [17.43–106.35]
***UGT1A4 Pro24Thr*** 24Thr/24Thr (n = 1)	16.93 ± 0.00 *16*.*93*	3.52 ± 0.00 *3*.*52*	10.80 ± 0.0 *10*.*8*	396.73 ± 0.00 *396*.*73*	8.01 ± 0.00 *8*.*01*	782.92 ± 0.00 *782*.*92*	30.30 ± 0.00 *30*.*30*	68.00 ± 0.00 *68*.*00*
***P value***	0.376	0.352	0.506	0.111	0.694	0.288	0.618	0.288
***UGT1A4 Leu48Val*** wt/wt (n = 117)	21.51± 16.67 *17*.*81* [0.00–153.88]	5.42 ± 4.66 *4*.*64* [0.00–34.42]	20.78 ± 14.90 *17*.*73* [0.00–73.72]	207.98 ± 97.88 *184*.*21* [77.10–576.23]	8.06 ± 7.57 *6*.*05* [0.00–35.90]	457.98 ± 198.55 *418*.*46* [35.96–1,210.09]	25.55 ± 20.33 *18*.*91* [0.00–107.10]	51.12 ± 23.42 *48*.*81* [15.69–142.97]
***UGT1A4 Leu48Val*** wt/48Val (n = 11)	21.07 ± 10.87 *19*.*04* [6.13–41.62]	4.65 ± 3.91 *4*.*58* [0.00–12.60]	23.48 ± 21.87 *16*.*60* [0.00–79.93]	178.21 ± 72.97 *153*.*75* [91.96–334.28]	8.06 ± 5.09 *8*.*30* [0.00–16.29]	415.65 ± 121.96 *448*.*61* [167.40–613.04]	19.26 ± 8.60 *20*.*26* [5.13–31.21]	49.16 ± 26.64 *42*.*72* [19.18–99.37]
***UGT1A4 Leu48Val*** 48Val/48Val (n = 2)	18.13 ± 1.24 *18*.*13* [17.25–19.01]	0.00 ± 0.00 *0*.*00* [0.00–0.00]	3.75 ± 5.31 *3*.*76* [0.00–7.51]	88.08 ± 0.00 *88*.*08* [88.08–88.08]	0.00 ± 0.00 *0*.*00* [0.00–0.00]	319.28 ± 0.00 *319*.*28* [319.28–319.28]	4.46 ± 0.00 *4*.*46* [4.46–4.46]	24.40 ± 0.00 *24*.*40* [24.40–24.40]
***P value***	0.966	0.130	0.128	0.148	0.204	0.728	0.246	0.333
***UGT2B7 His268Tyr*** wt/wt (n = 75)	21.45 ± 19.02 *16*.*73* [0.00–153.88]	4.87 ± 3.61 *4*.*37* [0.00–15.87]	20.35 ± 14.68 *16*.*60* [0.00–79.93]	188.98 ± 92.07 *167*.*01* [77.10–573.83]	6.70 ± 6.89 *5*.*69* [0.00–35.90]	416.94 ± 189.52 *372*.*61* [35.96–1,191.13]	23.61 ± 17.60 *18*.*79* [0.00–95.87]	47.66 ± 23.95 *41*.*84* [15.69–142.97]
***UGT2B7 His268Tyr*** wt/268Tyr (n = 22)	22.63 ± 11.21 *20*.*90* [4.81–45.21]	5.85 ± 5.20 *4*.*40* [0.00–18.97]	22.45 ± 18.29 *18*.*01* [0.00–71.00]	209.20 ± 82.63 *187*.*36* [102.43–378.72]	7.02 ± 5.61 *5*.*99* [0.00–26.98]	476.81 ± 172.86 *472*.*41* [212.67–952.85]	19.06 ± 13.47 *14*.*01* [5.01–51.89]	54.13 ± 25.03 *52*.*30* [17.43–106.35]
***UGT2B7 His268Tyr*** 268Tyr /268Tyr (n = 28)	20.30 ± 10.07 *18*.*64* [3.88–37.76]	6.25 ± 6.37 *4*.*71* [0.00–34.42]	21.59 ± 16.54 *18*.*02* [0.00–73.72]	238.81 ± 116.07 *203*.*66* [103.47–576.23]	11.37 ± 8.08 *8*.*72* [0.00–30.96]	526.70 ± 211.73 *487*.*68* [301.70–1,210.09]	33.22 ± 27.00 *24*.*65* [4.57–107.10]	55.31 ± 22.98 *52*.*30* [15.69–106.35]
***P value***	0.418	0.643	0.921	0.049	0.006	0.012	0.136	0.174
***UGT2B15 Asp85Tyr*** wt/wt (n = 39)	20.83 ± 11.21 *17*.*99* [4.81–62.07]	5.05 ± 4.01 *4*.*25* [0.00–15.87]	22.36 ± 18.35 *17*.*73* [0.00–79.93]	193.82 ± 97.03 *167*.*01* [89.79–576.23]	7.86 ± 7.30 *5*.*70* [0.00–30.96]	448.63 ± 206.96 *364*.*81* [242.88–1,210.09]	26.01 ± 21.93 *19*.*32* [0.00–95.87]	47.11 ± 23.69 *40*.*10* [15.69–106.35]
***UGT2B15 Asp85Tyr*** wt/85Tyr (n = 58)	23.47 ± 21.12 *18*.*56* [0.00–153.88]	5.41 ± 5.22 *4*.*91* [0.00–34.42]	20.67 ± 15.21 *18*.*05* [0.00–73.72]	212.51 ± 105.11 *184*.*50* [77.10–573.83]	8.53 ± 8.05 *6*.*60* [0.00–35.90]	446.42 ± 193.80 *398*.*77* [167.40–1,191.13]	25.06 ± 19.90 *19*.*93* [4.46–107.10]	53.15 ± 24.44 *50*.*56* [15.69–142.97]
***UGT2B15 Asp85Tyr*** 85Tyr/85Tyr (n = 35)	19.58 ± 10.17 *18*.*09* [0.00–47.54]	5.09 ± 4.17 *4*.*64* [0.00–18.97]	18.46 ± 12.75 *16*.*04* [0.00–49.99]	195.97 ± 78.13 *169*.*73* [102.91–378.32]	6.89 ± 6.14 *5*.*12* [0.00–26.98]	461.91 ± 175.10 *455*.*07* [35.96–952.85]	22.62 ± 16.21 *16*.*78* [4.03–53.75]	50.17 ± 21.69 *45*.*33* [22.66–106.35]
***P value***	0.810	0.895	0.778	0.633	0.709	0.547	0.720	0.311
***UGT2B15 Lys523Thr*** wt/wt (n = 37)	22.06 ± 24.58 *15*.*92* [0.00–153.88]	3.91 ± 3.70 *3*.*52* [0.00–18.97]	15.36 ± 11.66 *14*.*80* [0.00–49.99]	198.25 ± 103.42 *171*.*97* [77.10–576.23]	6.86 ± 8.24 *4*.*68* [0.00–30.96]	448.88 ± 213.60 *396*.*18* [196.74–1,210.09]	20.25 ± 17.12 *16*.*39* [0.00–89.98]	49.28 ± 21.69 *43*.*59* [20.53–106.35]
***UGT2B15 Lys523Thr*** wt/523Thr (n = 68)	21.46 ± 12.40 18.62 [0.00–64.51]	5.28 ± 3.71 *5*.*01* [0.00–15.83]	21.71 ± 16.29 *18*.*31* [0.00–79.93]	200.38 ± 83.50 *184*.*89* [88.08–515.07]	7.50 ± 5.58 *6*.*36* [0.00–27.86]	458.19 ± 163.84 *454*.*91* [212.67–968.42]	24.88 ± 17.96 *21*.*05* [4.18–107.10]	49.52 ± 21.50 *48*.*81* [15.69–111.58]
***UGT2B15 Lys523Thr*** 523Thr /523Thr (n = 22)	20.15 ± 7.70 *18*.*48* [9.62–37.68]	5.40 ± 4.16 *4*.*48* [0.00–15.87]	19.87 ± 12.12 *19*.*76* [0.00–51.78]	216.42 ± 116.89 *176*.*45* [91.96–573.83]	10.07 ± 9.21 *6*.*58* [0.00–35.90]	450.96 ± 248.30 *409*.*42* [35.96–1,191.13]	28.72 ± 25.92 *19*.*12* [5.40–95.87]	55.38 ± 32.17 *45*.*33* [15.69–142.97]
***P value***	0.472	0.070	0.082	0.809	0.128	0.637	0.299	0.930
***UGT2B17*** wt/wt (n = 75)	22.58 ± 18.79 *18*.*49* [0.00–153.88]	5.07 ± 4.78 *4*.*64* [0.00–34.42]	19.28 ± 14.77 *16*.*34* [0.00–73.72]	203.09 ± 95.32 *184*.*50* [89.79–576.23]	7.34 ± 6.74 *5*.*82* [0.00–30.96]	457.24 ± 179.79 *421*.*86* [238.36–1,210.09]	24.69 ± 19.96 *18*.*69* [0.00–107.10]	48.89 ± 20.72 *44*.*46* [15.69–106.35]
***UGT2B17*** wt/del (n = 44)	18.75 ± 11.29 *16*.*29* [0.00–64.51]	5.33 ± 4.34 *4*.*16* [0.00–15.87]	22.36 ± 17.73 *18*.*44* [0.00–79.93]	204.70 ± 106.03 *175*.*91* [77.10–573.83]	9.61 ± 8.56 *7*.*56* [0.00–35.90]	460.67 ± 223.90 *389*.*90* [167.40–1,191.13]	27.02 ± 21.09 *22*.*58* [4.03–95.87]	53.00 ± 28.85 *45*.*33* [15.69–142.97]
***UGT2B17*** del/del (n = 12)	26.96 ± 12.38 *25*.*11* [5.95–47.54]	5.53 ± 4.84 *5*.*03* [0.00–18.97]	20.77 ± 12.51 *19*.*77* [0.00–49.99]	203.34 ± 71.08 *178*.*70* [130.69–375.04]	5.28 ± 5.09 *4*.*86* [0.00–16.40]	410.68 ± 144.70 *473*.*48* [35.96–545.30]	16.55 ± 8.88 *15*.*98* [6.19–38.14]	51.40 ± 17.89 *54*.*92* [22.66–78.45]
***P value***	0.042	0.934	0.646	0.846	0.141	0.928	0.325	0.796

## Discussion

The prevalences of *UGT* genotypes and alleles observed in our study are in good agreement with those reported for other European populations [18, 20, 22, 31, 33]. The enzyme UGT1A4, besides giving rise to glucuronidated TAM metabolites, also catalyzes the formation of a variety of carcinogenic compounds, androgens, progestins and plant steroids [15, 16, 20] and shows a substrate-dependent glucuronidation efficacy. In *in vitro* studies, similar TAM glucuronidation rates have been observed for both mutant variants (UGT1A4^24Thr^ and UGT1A4^48Val^) and the wild type enzyme [11, 19]. Consistently, we detected similar glucuronidation activity *in vivo* for both *UGT1A4* ^*24Thr*^ and *UGT1A4* ^*48Val*^ when the genotypes were considered separately (Table 5). However, when genotypes were grouped, homozygous mutant *UGT1A4* ^*48Val*^ patients showed significantly lower concentrations of both 4-OH-TAM-O-Gluc and endoxifen-Gluc versus wt/wt plus wt/48Val patients (n = 128) (p = 0.037, p = 0.031, respectively, Fig 1). These data suggest that the *UGT1A4* ^*Leu48Val*^ polymorphism could be correlated with significantly reduced glucuronidation levels of active hydroxylated TAM metabolites, and has been attributed similar effects on substrates such as naphthylamine or dihydrotestosterone [18]. Similar findings have been recently reported for 4-OH-TAM in microsomes in the case of other SNPs affecting the *UGT1A4* promotor region [35]. Modified glucuronidation could affect half-lives of circulating TAM metabolites and this could alter the effectiveness of these drugs for the treatment of breast cancer. Further, when TAM concentrations were measured in homozygous mutant *UGT1A4* ^*48Val*^ individuals, these were found to be reduced compared with the levels detected in subjects with the wt/wt or wt/48Val genotypes (not shown). Although we could not determine TAM-Gluc levels, these results suggest greater enzyme activity against the TAM substrate in patients with two mutant 48Val alleles. Mürdter et al. [28] confirmed this hypothesis of the role of *UGT1A4* in the formation of the N-glucuronide of TAM in that correlation was detected between the variant 48Val codon and higher TAM-N-Gluc levels. These authors noted a significantly lower metabolic ratio TAM/TAM-N-Gluc in patients with two *UGT1A4* ^*48Val*^ alleles. This genotype is known to enhance glucuronidation activity against TAM as compared to the wild type *UGT1A4* ^*48Leu*^ variant.

This study is the first to quantify plasma glucuronide TAM metabolites in individuals with *del UGT2B17* genotypes. So far, effects of the presence of a *UGT2B17* deletion have been examined on the substrates oxazepam and androgenic steroids, with no modifications produced in the availability of both drugs [23, 24]. In our study, mean 4-OH-TAM-N-Gluc levels varied significantly among the three *UGT2B17* genotypes (p = 0.042, Table 5) while levels were significantly higher when del/del was compared to wt/wt plus wt/del (p = 0.036, Fig 1). In addition, patients in the *UGT2B17* del/del group (n = 12) showed a significantly lower mean 4-OH-TAM/4-OH-TAM-N-Gluc ratio versus the remaining patients (0.20 ± 0.18 and 0.5 ± 0.73, p = 0.028). These conflicting results could be explained by a compensatory mechanism inducing the upregulation of other genes in del/del *UGT2B17* individuals to produce higher concentrations of glucuronide products. When examining testosterone metabolism, Schulze et al. [36] proposed that a lack of the UGT2B17 enzyme could be offset by augmented *UGT2B15* transcription. These authors found that individuals homozygous for the *UGT2B17* deletion showed liver *UGT2B15* mRNA levels that were 4.5-fold the levels observed in individuals carrying two functional *UGT2B17* alleles. This observation is consistent with a duplication event as the origin of these genes [21]. However, we could not examine this issue here because of the low number of individuals with the del/del genotype (n = 12). Opposing results have been reported for other substrates. For instance, individuals carrying the *UGT2B17* deletion have been described to show both significantly reduced overall glucuronidation rates of nicotine and its major metabolites in smokers [37] and of androgen substrates in prostate cancer patients [38].

*UGT2B15* ^*Asp85Tyr*^ did not appear to determine interindividual variation in TAM metabolites since no significant differences were observed among genotypes in plasma metabolite concentrations (Table 5). Similarly, Mürdter et al. [28] detected no correlation between this polymorphism and both hydroxylated TAM metabolites and their corresponding glucuronide products. *UGT2B15* ^*Asp85Tyr*^ has been nevertheless identified as major determinant of glucuronidation by the human liver of other substrates suggesting a substrate-dependent effect of this polymorphism. For example, the *UGT2B15* ^*85Tyr*^ variant has been related both to reduced oxazepam [23, 27] and lorazepam [39] glucuronidation and enhanced 19-norandrosternone glucuronidation [40]. In the case of *UGT2B15* ^*Lys523Thr*^, we here submit the first data indicating a variant gene-dose effect in that significantly higher levels of 4-OH-TAM-O-Gluc and endoxifen-Gluc (0.023 and 0.025, respectively, Fig 2) were detected in women with at least one variant *UGT2B15* ^*523Thr*^ allele *versus* women with the wt/wt genotype. Such higher glucuronidation activity related to *UGT2B15* ^*523Thr*^ was also detected by Court et al. [27] against oxazepam in HLM from male heterozygotes compared with wild type homozygotes, although others have reported no such correlation of this polymorphism with oxazepam availability [23].

*UGT2B7* genotypes showed significant differences in plasma levels of TAM, 4-OH-TAM and N- desmethyl-TAM (p = 0.049, p = 0.006 and p = 0.012, respectively, Table 5) indicating possible lower glucuronidation activity in these individuals. When genotypes were grouped by mutant allele dose, differences in these metabolites were also significant (Fig 2). This is the first description of this link in an *in vivo* study. Other authors have also described a decreasing trend in O-glucuronidation of 4-OH-TAM with increasing numbers of *UGT2B7* ^*268Tyr*^ alleles in HLM [30]. Similar observations have been made in cell lines whereby the *UGT2B7* ^*268Tyr*^ variant was associated with significant 2- and 5-fold decreases in activity against 4-OH-TAM and endoxifen respectively compared with wild-type *UGT2B7* ^*268His*^ [29]. Wiener et al. [41] also demonstrated lower UGT2B7 activity against the substrate butanone associated with homozygous mutant microsomes. In contrast, Mürdter et al. [28] detected no correlations between plasma concentrations of endoxifen or 4-OH-TAM and *UGT2B7* ^*268Tyr*^ and these findings were consistent with the metabolic ratios of their glucuronidation reactions. In our study, the substrate/product ratios that could be calculated, 4-OH-TAM/4-OH-TAM-O-Gluc and 4-OH-TAM/4-OH-TAM-N-Gluc also indicated the reduced activity of the enzyme for the mutant *UGT2B7* ^*268Tyr*^ allele. One of the limitations of our study was the sample size, although the number of patients examined was similar to those included in other studies [42, 43, 44]. The small numbers of some genotypes compared will have led to a low statistical power in some of the tests. It could therefore be that other genotype effects would have emerged if we had had data for a larger patient cohort. This issue should be resolved in future studies.

In a prior study, we noted that besides the *CYP2D6* genotype leading to tamoxifen conversion to potent hydroxylated metabolites in a manner consistent with a gene-dose effect, *SULT1A2* also seems to play a role in maintaining optimal levels of both 4-hydroxy-tamoxifen and endoxifen. The present paper presents the first data on a possible greater effect of the *UGT2B17del* and *UGT2B15* ^*523Thr*^ genotypes on TAM glucuronidation. In contrast, the variant alleles *UGT1A4* ^*48Val*^ and *UGT2B7* ^*268Tyr*^ seem to confer reduced glucuronidase activity. Thus patients carrying mutations *UGT1A4* ^*48Val*^ and *UGT2B7* ^*268Tyr*^ or with wild type *UGT2B17* ^*nodel*^ and *UGT2B15* ^*523Lys*^ genotypes could be the best candidates for a good response to TAM therapy in terms of inducing effective plasma active TAM metabolite levels. In summary, the results of this study suggest that genetic UGT variants that are highly active against TAM metabolites will significantly alter TAM metabolism and consequently its elimination in TAM-treated individuals. Similarly to that described above for *CYP2D6* and *SULT1A2*, a woman's UGT genotype could affect her overall response to TAM. Additional studies examining the effects of UGT genotypes on overall patient response to TAM are needed to further examine the role of UGT polymorphisms in the therapeutic efficacy of TAM.
