# Correction: Impacts of the Glucuronidase Genotypes *UGT1A4*, *UGT2B7*, *UGT2B15* and *UGT2B17* on Tamoxifen Metabolism in Breast Cancer Patients

**DOI:** 10.1371/journal.pone.0140921

**Published:** 2015-10-23

**Authors:** The PLOS ONE Staff

The Funding statement is incorrect. The publisher apologizes for the error. The complete, correct Funding statement is: This work has been supported partially by a grant from Fundación de Investigación Médica Mutua Madrileña (ref number AP27072008), a grant from Universidad Europea de Madrid (project number 2013/UEM31) and by the help of Cátedra Florencio Tejerina-UEM in blood sample collection. The funders had no role in study design, data collection and analysis, decision to publish, or preparation of the manuscript.

## References

[1] Romero-Lorca A, Novillo A, Gaibar M, Bandrés F, Fernández-Santander A (2015) Impacts of the Glucuronidase Genotypes *UGT1A4*, *UGT2B7*, *UGT2B15* and *UGT2B17* on Tamoxifen Metabolism in Breast Cancer Patients. PLoS ONE 10(7): e0132269 doi: 10.1371/journal.pone.0132269 26176234 10.1371/journal.pone.0132269 PMC4503404

